# Peripheral Interaction of Resolvin D1 and E1 with Opioid Receptor Antagonists for Antinociception in Inflammatory Pain in Rats

**DOI:** 10.3389/fnmol.2017.00242

**Published:** 2017-08-03

**Authors:** Beatrice Oehler, Milad Mohammadi, Cristina Perpina Viciano, Dagmar Hackel, Carsten Hoffmann, Alexander Brack, Heike L. Rittner

**Affiliations:** ^1^Department of Anesthesiology and Critical Care, University Hospital of Wuerzburg Wuerzburg, Germany; ^2^Bio-Imaging-Center/Rudolf-Virchow-Center, Institute of Pharmacology, University of Wuerzburg Wuerzburg, Germany; ^3^Institute for Molecular Cell Biology, CMB—Center for Molecular Biomedicine, University Hospital Jena Jena, Germany

**Keywords:** resolvin, opioid receptors, opioid peptides, transient receptor potential channels, inflammation, animals, pain behavior

## Abstract

Antinociceptive pathways are activated in the periphery in inflammatory pain, for instance resolvins and opioid peptides. Resolvins are biosynthesized from omega-3 polyunsaturated fatty acids such as eicosapentaenoic acid and docosahexaenoic acid. Resolvin D1 (RvD1) and resolvin E1 (RvE1) initiate the resolution of inflammation and control of hypersensitivity via induction of anti-inflammatory signaling cascades. RvD1 binds to lipoxin A4/annexin-A1 receptor/formyl-peptide receptor 2 (ALX/FPR2), RvE1 to chemerin receptor 23 (ChemR23). Antinociception of RvD1 is mediated by interaction with transient receptor potential channels ankyrin 1 (TRPA1). Endogenous opioid peptides are synthesized and released from leukocytes in the tissue and bind to opioid receptors on nociceptor terminals. Here, we further explored peripheral mechanisms of RvD1 and chemerin (Chem), the ligand of ChemR23, in complete Freund’s adjuvant (CFA)-induced hindpaw inflammation in male Wistar rats. RvD1 and Chem ameliorated CFA-induced hypersensitivity in early and late inflammatory phases. This was prevented by peripheral blockade of the μ-opioid peptide receptor (MOR) using low dose local naloxone or by local injection of anti-β-endorphin and anti-met-enkephalin (anti-ENK) antibodies. Naloxone also hindered antinociception by the TRPA1 inhibitor HC-030031. RvD1 did not stimulate the release of β-endorphin from macrophages and neutrophils, nor did RvD1 itself activate G-proteins coupled MOR or initiate β-arrestin recruitment to the membrane. TRPA1 blockade by HC-030031 in inflammation *in vivo* as well as inhibition of the TRPA1-mediated calcium influx in dorsal root ganglia neurons *in vitro* was hampered by naloxone. Peripheral application of naloxone alone *in vivo* already lowered mechanical nociceptive thresholds. Therefore, either a perturbation of the balance of endogenous pro- and antinociceptive mechanisms in early and late inflammation, or an interaction of TRPA1 and opioid receptors weaken the antinociceptive potency of RvD1 and TRPA1 blockers.

## Introduction

Resolvins are lipid mediators that rise during resolution of acute inflammation from poly-unsaturated fatty acids and docosahexanoic acid (Serhan, 2014). Resolvins such as resolvin D1 (RvD1) and resolvin E1 (RvE1) facilitate the resolution of inflammation (Ji et al., 2011) in, for example, sepsis, asthma, atherosclerosis, and osteoarthritis (Merched et al., 2008; Xu and Ji, 2011; Chiang et al., 2015; Provoost et al., 2016; Huang et al., 2017). All known resolvin receptors are G protein-coupled receptors (GPCR). They interfere with immune cell functions to promote resolution. RvD1 binds to the lipoxin A4/annexin-A1 receptor/formyl-peptide receptor 2 (ALX/FPR2) as well as to the G-protein-coupled receptor 32 (GPR32; Krishnamoorthy et al., 2010). RvE1 and chemerin (Chem), an endogenous peptide agonist, bind to the Chem receptor 23 (ChemR23; Arita et al., 2005). Intracellular pro-inflammatory signaling cascades are downregulated by resolvin receptor activation. Chem, a 14 kDa protein secreted in an inactive form as pro-Chem and activated through cleavage of the C-terminus by serine proteases as activated in inflammation and coagulation (Xu et al., 2010).

Transient receptor potential channels of the ankyrin 1 (TRPA1) and vanilloid subtype (TRPV1) are chemosensors in nociceptors (Gangadharan and Kuner, 2013). TRPV1 is activated by heat (>42°C), acidosis, endogenous lipidergic pro-inflammatory messengers such as leukotrienes and natural pungent compounds such as capsaicin (Tominaga et al., 1998). TRPA1 responds to exogenous irritants like allyl isothiocyanate (AITC; Bautista et al., 2006) and endogenous lipids like oxidized phospholipids found in complete Freund’s adjuvant (CFA)-induced hindpaw inflammation.

Resolvins were the first endogenous TRPA1 and TRPV1 channel inhibitors described (Park et al., 2011b; Serhan, 2014; Lim et al., 2015). Apart from their inflammation resolving properties, resolvins have antinociceptive properties: intraplantar and intrathecal application of RvD1 and RvE1 reduce thermal hypersensitivity and local inflammation induced by carrageenan, CFA, or formalin without changing basal nociceptive thresholds (Xu et al., 2010). Intrathecal administration of RvD1 before surgery increases nociceptive thresholds. When administered on the first postoperative day, animals showed the same pain as before surgery (Huang et al., 2011). Beside of TRPA1 and TRPV1, antinociception by RvD1 is mediated by inhibition of TRPV3 and TRPV4, also expressed in peripheral nerve endings in the skin and muscles (Bang et al., 2010; Xu et al., 2010). Specifically, spinal opioid receptors are not involved in RvE1-evoked antinociception (Xu et al., 2010; Park et al., 2011a).

In the early phase of inflammation, neutrophils are the predominant leukocyte population, during the late phase, macrophages are more prevalent (Brack et al., 2004). Endogenous opioids like met-enkephalin and β-endorphin are produced and released from neutrophils and monocytes/macrophages (Rittner et al., 2001, 2006, 2009; Sauer et al., 2014; Wang et al., 2014). They bind to the G-protein-coupled μ-opioid receptor (MOR) expressed in the peripheral nociceptive system (Mambretti et al., 2016; Corder et al., 2017; Spahn et al., 2017). MOR activates several signaling cascades including G_i/o_-protein-dependent and -independent signaling pathways such as activation of adenylate cyclase, phosphorylation by protein kinases, activation of specific potassium calcium channels, and β-arrestin recruitment (Mambretti et al., 2016). Naloxone, a competitive antagonist of MOR, as well as antibodies against endogenous opioid peptides like anti-met-enkephalin (anti-ENK) or anti-β-endorphin (anti-END)—applied locally—prevent e.g., formyl-peptide-induced antinociception (Rittner et al., 2009; Wang et al., 2014).

In our study here, we further investigated peripheral antinociceptive properties of RvD1 and Chem, an agonist to the same receptor as RvE1, in rats in the early and late phase of local inflammation and deciphered its local mechanism. We hypothesized that RvD1 and Chem interact with opioid receptor activation on peripheral nerve terminals and/or TRPA1 activation.

## Materials and Methods

### Reagents and Chemicals

The following reagents were used: AITC, HC-030031, naloxone, DAMGO, IgG, fMLP and ionomycin (Sigma-Aldrich, Taufkirchen, Germany), RvD1 (Cayman, Ann Arbor, MI, USA), Chem (R&D Systems Inc., Minneapolis, MN, USA), anti-END and anti-ENK (Bachem, Weil am Rhein, Germany), anti-PMN serum (Accurate Chemical and Scientific Corporation, Westbury, NY, USA), and CFA (Calbiochem, San Diego, USA/BD Bioscience, San Jose, CA, USA). Dimethyl sulfoxide or aqueous physiological solutions served as solvents.

Chemicals and reagents for cell culture experiments included gentamycin, penicillin, streptomycin, nerve growth factor (NGF), IST liquid media supplement (100×), HEPES (4-(2-hydroxyethyl)piperazine-1-ethanesulfonic acid, N-(2-Hydroxyethyl)piperazine-N′-(2-ethanesulfonic acid)), poly-L-lysine, DMEM/F12, fetal bovine serum (Life Technologies GmbH, Darmstadt, Germany), and Effectene transfection reagent (Qiagen, Hilden, Germany).

### *In Vivo* Studies

#### Animals

This study was carried out in accordance with the recommendations of International Association for the Study of Pain (IASP). The protocol was approved by the animal care committee of the provincial government of Wuerzburg (55.2-2531.01-5/13). Male Wistar rats weighing 180–220 g were injected intraplantarly under brief isoflurane anesthesia as described below. Animals were randomly assigned to treatments by numbers within a cage.

#### Measurement of Nociceptive Thresholds

Mechanical thresholds were determined using the paw pressure algesiometer (modified Randall-Selitto test; Ugo Basile, Comerio, Italy; Hackel et al., 2012). The pressure required to elicit paw withdrawal (paw pressure threshold, PPT) was determined by a blinded investigator. Averages from three measurements per treatment were calculated. Baseline measurements were obtained before and 2 h or 96 h after intraplantar injection of 150 μl CFA. After indicated time points, RvD1, Chem, or HC-030031 dissolved in 150 μl 0.9% saline were applied. PPT were determined 15, 45 and 180 min thereafter. In selected experiments, 0.56 ng naloxone (NLX), anti-END (2 μg) or anti-ENK (1.25 μg; Rittner et al., 2006) dissolved in 0.9% saline were injected intraplantarly before or together with TRP channel antagonists, RvD1 or Chem. Anti-PMN (80 μl) was injected i.p. 15–18 h before CFA to deplete neutrophils (Rittner et al., 2006). Doses were chosen based on pilot experiments and on the literature (Xu et al., 2010; Liu et al., 2016).

### *In Vitro* Studies

#### Primary Culture of Dorsal Root Ganglia Neurons (DRG)

Preparation of Dorsal Root Ganglia (DRG) from adult wildtype mice were carried out as described (Schulze et al., 2013). DRG neurons were grown at a density of 7 × 10^3^ cells per glass cover slip coated with poly-L-lysine (20 μg/ml) and cultured at 37°C, 5% CO_2_ atmosphere for 1 day. Measurements were performed the following day. Medium contained 100 ng/ml NGF (Sigma-Aldrich, Taufkirchen, Germany).

#### Calcium Imaging

For ratiometric single cell calcium analysis, DRGs were labeled with Fura-2/AM in imaging solution (in mM): 134 NaCl, 6 KCl, 1 MgCl_2_, 1 CaCl_2_, 10 HEPES, 5.5 glucose, pH 7.4 adjusted with NaOH (Oehler et al., 2012, 2017). All measurements were performed at room temperature using a Nikon TE2000-E microscope. Fura-2/AM was excited with a Lambda DG4/17 wavelength switch (Sutter Instruments, Novato, CA, USA). Time-lapse image series were acquired with a cooled EMCCD Andor iXon camera (Andor Technology Ltd., Belfast, UK) controlled by NIS Elements Software (Nikon, Düsseldorf, Germany). Objective: CFI S-Fluor 10×/0.5 (Nikon). Image series were analyzed with ImageJ 1.46r, time series analyzer V2.0 plugin (Rasband, W.S., ImageJ, U.S. National Institutes of Health, Bethesda, MD, USA). AITC was used as TRPA1 agonist and β-endorphin as MOR agonist. The mean of basal fluorescence intensity was determined for each measurement. Number of reacting cells (%) was calculated by 1.5-fold increase of mean basal fluorescent intensity after stimulation. The area under curve (AUC) was taken from the mean of five individual experiments. Intervals correspond to the stimulation period of AITC.

### β-Endorphin Release

Rat neutrophils were isolated by lavage of the peritoneal cavity with 2 mM EDTA/phosphate buffered saline (PBS) 4 h after i.p. injection of 20 ml 1% oyster glycogen (Rittner et al., 2006), macrophages 4 days after i.p. injection of 3% thioglycollate (Hackel et al., 2013). Neutrophils or macrophages (10^7^ cells in Hanks balanced salt solution (HBSS)) were treated with cytochalasin B (5 μg/ml) at 37°C for 5 min for preactivation (Rittner et al., 2009). Subsequently, RvD1 or ionomycin (10 μM) and fMLP (1 μM) dissolved in HBSS containing bestatin, aprotinin, and thiorphan, was added. To terminate stimulation, cells were cooled on ice. β-endorphin was measured in the supernatant (β-endorphin (rat) EIA Kit, Phoenix Pharmaceuticals, Burlingame, CA, USA)). Absorbance was measured at 450 nm (Magellan V5.01 software, Tecan, Crailsheim, Germany).

### Kinetic Measurements of Gαi Activation

HEK-293 cells were cultured in DMEM, high glucose, 10% fetal calf serum (Biochrom, Berlin, Germany), 100 Uml^−1^ penicillin G, and 100 μgml^−1^ streptomycin sulfate. For kinetic measurements of Gαi1, Gαi2 and Gαi3 activation, HEK-293 cells were seeded on 40 mm poly-D-lysin-coated plates 1 day before transfection. Transient transfection with 0.63 μg of untagged MOR and 0.63 μg of the corresponding subtype of G protein sensor, either Gαi1, Gαi2 or Gαi3 (pGβ1-2A-yellow fluorescent protein (YFP)-Gγ2-IRES-Gαi-mTq2 cDNA; van Unen et al., 2016) per dish and Effectene transfection reagent (Qiagen, Hilden, Germany) was performed according to manufacturer’s instructions. FRET-measurements were accomplished the next day. During the experiment, cells were superfused with measuring buffer (140 mM NaCl, 5.4 mM KCl, 2 mM CaCl_2_, 1 mM MgCl_2_, 10 mM HEPES, pH 7.3) supplemented with the ligand RvD1 or DAMGO at the indicated concentration at indicated time points, using a microfluidic pipette (Ainla et al., 2012; Fluicell, Gothenburg, Sweden).

Förster resonance energy transfer (FRET) measurements were performed as previously described (Hoffmann et al., 2005) with slight modifications. In brief, FRET measurements were performed on an inverted microscope (Zeiss Axiovert 200, Zeiss, Jena, Germany) equipped with an oil immersion 63× objective lens and a dual-emission photometric system (Till Photonics, Gräfelfing, Germany). The transfected cells were excited with light from a polychrome IV (Till Photonics) at a frequency of 10 Hz with 20 ms illumination out of a total time of 100 ms. Emission of cyan fluorescent protein (CFP, 480 ± 20 nm) and YFP (535 ± 15 nm), and the FRET ratio (FYFP/FCFP) were monitored simultaneously (beam splitter DCLP 505 nm) upon excitation at 436 ± 10 nm (beam splitter DCLP 460 nm). Fluorescence signals were detected by photodiodes, digitalized using an analog-digital converter (Digidata 1440A, Axon Instruments, Sunnyvale, CA, USA) and stored with Clampex 9.0 software (Science Products, Hofheim, Germany). Processing of the data was done in Origin 2017 (Additive, Friedrichsdorf, Germany) and GraphPad Prism (La Jolla, CA, USA).

### β-Arrestin-2 Recruitment Assays

HEK-293 cells were seeded on 24 mm glass coverslips coated with poly-D-lysine for 20 min, placed in 6-well plates. Twenty-four hours later, cells were transiently transfected with three plasmids using Effectene: 2.6 μg of human MOP-CFP, 0.5 μg of β-arrestin-2-YFP, and 1.0 μg of GRK2 per dish (Hoffmann et al., 2008; Mambretti et al., 2016). All constructs were in pcDNA3. The day after, the medium was changed and cells analyzed 48 h after transfection.

The experiment was performed in a Leica TCS SP2 scanning microscope (Leica, Wetzlar, Germany) as previously described (Hoffmann et al., 2005). Images were taken with a HCX PL APO CS 63× 1.32 oil objective. In brief, CFP was excited with using 458 nm, and emission was detected from 465–500 nm while YFP was excited with the 514 nm line of the argon laser, and emission was detected from 525–600 nm. The following settings were kept constant over all the measurements: 512 × 512-pixel format, line average 2, frame average 2400 Hz. Pictures (time series) were taken at 30 s intervals for 20 or 30 min. MOP-CFP was useful to specifically target cells which express the receptor in the membrane.

β-arrestin translocation to the membrane was quantified using Leica Confocal SP2 Software. Regions of interest, ROIs, were selected in the cytosol of the cells and quantified over time. In order to correct for photobleaching, control regions were selected (whole cells) and quantified too. Upon correction, the fluorescence intensity values were normalized to the value right before the recruitment takes place and plotted against time.

### Statistical Analysis

*In vivo* data are presented as mean ± SEM. Either two-way ANOVA with repeated measurements (behavior experiments), one-way ANOVA (β-endorphin release) or student’s *t*-tests (calcium imaging) were performed with SigmaPlot12 or Origin 2017.

## Results

### Local Antinociception of RvD1 and Chem in Early and Late CFA-Induced Hind Paw Inflammation

**Figure 1:**
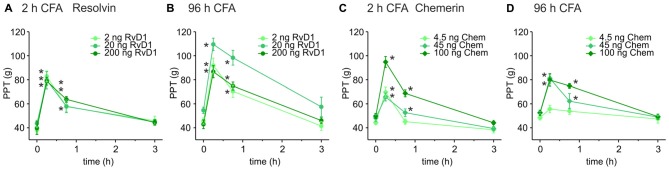
Antinociception by resolvin D1 (RvD1) and chemerin (Chem) in early and late states of complete freund’s adjuvant (CFA)-induced hind paw inflammation in rats. Antinociceptive effects of RvD1 **(A,B)** and Chem **(C,D)** were measured in the early (2 h; **A,C**) and the late phase (96 h; **B,D**) of CFA-evoked hind paw inflammation in male Wistar rats. Mechanical hypersensitivity (paw pressure thresholds (PPTs); in g) was evaluated at indicated time points after intraplantar injection of RvD1 or Chem. Baseline values were obtained before injection of RvD1 or Chem. Dose-dependency was evaluated by injection of three different concentrations of RvD1 and Chem (see legends; *n* = 4–6 per group, mean ± SEM, two-way ANOVA RM *post hoc* Holm-Sidak, *p < 0.05 vs. baseline pre-injection values, 0 h).

Intrathecal applications of RvD1 and Chem reduce nociceptive behavior after formalin injection and thermal hypersensitivity in short-term (2–4 h) carrageenan-induced hindpaw inflammation (Xu et al., 2010). Here, we asked whether these lipid mediators also act peripherally on sustained mechanical inflammatory hypersensitivity in a prolonged inflammatory model (CFA-induced hindpaw inflammation). Mechanical nociceptive thresholds decreased 2 h and 96 h after intraplantar CFA injection when RvD1 and Chem were applied intraplantarly at 2 h (Figures 1A,C) and 96 h (Figures 1B,D) after CFA application, respectively. PPTs were determined 15, 45 and 180 min after intraplantar antagonist injection. Both, RvD1 and Chem significantly reduced CFA-induced mechanical hypersensitivity. The highest concentration of Chem (100 ng) and 20 ng RvD1 were most effective in early as well as in late CFA-induced inflammation. Antinociception lasted for up 45 min and returned to baseline hypersensitivity after 3 h. Higher doses of RvD1 did not results in more effective or longer antinociception of RvD1. No change was seen in the contralateral paw (data not shown).

### Opioid Receptor and Ligand Participation in RvD1- or Chem-Generated Antinociception

**Figure 2:**
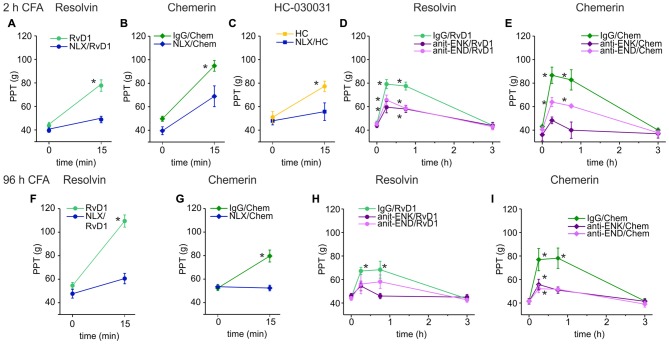
Reversal of RvD1-mediated antinociception by peripheral blockade of the μ-opioid peptide receptor (MOR) or of endogenous opioid peptides in rats. Antinociception by RvD1 **(A,D,F,H)**, Chem **(B,E,G,I)**, and HC-030031 (HC, **C**) in CFA-mediated hind paw inflammation on mechanical hypersensitivity was reduced by local co-application of naloxone **(A–C,F,G)** or local co-injection of antibodies against the endogenous opioid peptides β-endorphin (END) and met-enkephalin (ENK; anti-END/anti-ENK; **D,E,H,I**) at indicated time points in the early **(A–E)** and the late phase **(F–I)** of CFA-induced inflammation. Baseline inflammatory hypersensitivity was measured before injection of RvD1, Chem, or HC at CFA-time points indicated above the graphs (*n* = 6 per group, mean ± SEM; two-way ANOVA RM *post hoc* Holm-Sidak, *p < 0.05 compared to baseline pre-injection values, 0 h).

Next, we evaluated whether opioid receptors shape antinociception by RvD1 or Chem. Intraplantar injection of RvD1 or Chem together with naloxone either completely abolished or significantly reduced antinociception in early (Figures 2A,B) and late CFA-induced hind paw inflammation (Figures 2F,G). Since RvD1 is a TRPA1 antagonist, we replicated the experiment with the TRPA1 antagonist HC-030031 (Figure 2C). Similar to RvD1, intraplantar naloxone prevented the HC-030031-induced rise in mechanical nociceptive thresholds.

Peripheral opioid receptors are activated by release of endogenous opioid peptides secreted from leukocytes (Rittner et al., 2009). To define the contribution of endogenous ligands of MOR, antibodies against the opioid peptides anti-END and anti-ENK (Figures 2D,E,H,I), were injected locally together with RvD1 or Chem. IgG isotype antibodies served as controls to exclude unspecific effects. Both antibodies, anti-END and anti-ENK, significantly reduced RvD1- and Chem-mediated antinociception. In summary, peripheral opioid receptor activation by endogenous opioid peptides interacts with antinociceptive properties of RvD1, Chem and the TRPA1 antagonist HC-030031.

### Role of Leukocytes for RvD1-Mediated Peripheral Antinociception

**Figure 3:**
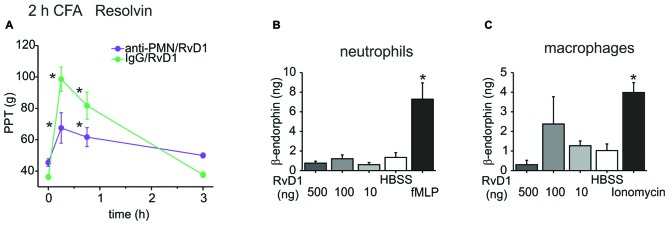
Leukocyte dependency of RvD1-mediated antinociception in early states of CFA-induced hind paw inflammation. Antinociception by RvD1 **(A)** was prevented in part in leukocyte-depleted Wistar rats. Leukocyte depletion was achieved by systemic anti-PMN (i.p.) treatment as described before (Rittner et al., 2006), IgG isotype antibody served as control. Mechanical hypersensitivity was measured at indicated time points before and after local RVD1 injection. (*n* = 4–6 per group, mean ± SEM; two-way ANOVA RM *post hoc* Holm-Sidak, *p < 0.05 compared to baseline pre-injection values, 0 h). **(B,C)** Release of β-endorphin from peritoneal neutrophils **(B)** and macrophages **(C)** of Wistar rats was not stimulated by different concentrations of RvD1. β-endorphin release (ng/ml per 1 × 10^7^ cells) was measured with a commercially available ELISA after stimulation of isolated cells by RvD1 at indicated concentrations. Hank’s balanced salt solution (HBSS) served as negative control, fMLP (**B**; 1 μM) or ionomycin (**C**; 10 μM) as positive control (*n* = 2–8 per group, mean ± SEM, one-way ANOVA *post hoc* Holm-Sidak, *p < 0.05 compared to HBSS solvent control).

To further investigate this pathway, we assessed the involvement of immune cells and their release of opioid peptides. In early CFA-induced (2 h) hind paw inflammation, systemic injection of an anti-polymorphonuclear neutrophil (anti-PMN) serum depleted neutrophils from the circulation and from the inflamed paw (Rittner et al., 2006, 2009). This treatment reduced antinociception evoked by RvD1 by 60% (Figure 3A). However, *in vitro* β-endorphin liberation from immune cells like rat peritoneal neutrophils or macrophages was not significantly increased after RvD1 stimulation independent of the dose (Figures 3B,C). As a positive control, stimulation with the chemotactic peptide, fMLP, or ionomycin lead to 2–4 times higher concentrations of released β-endorphin.

### No Direct Activation of MOR by RvD1

**Figure 4:**
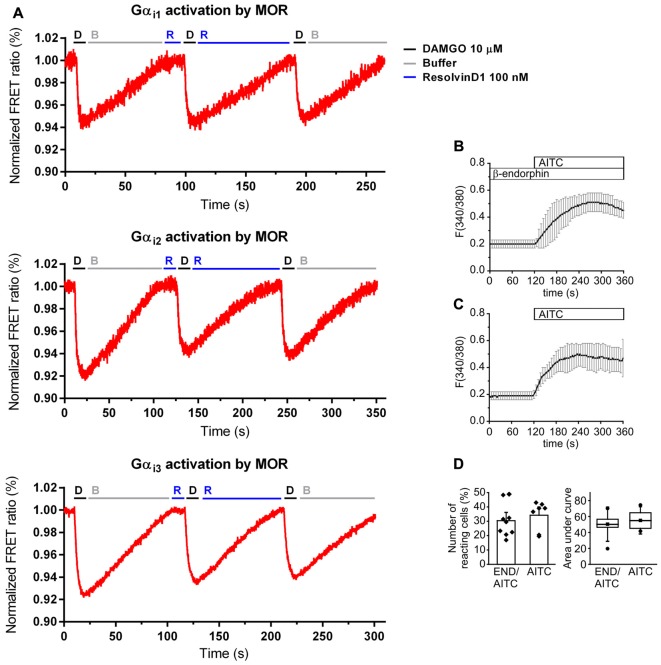
No activation of MOR by RvD1 in HEK-293 and lack of alteration of transient receptor potential channels ankyrin 1 (TRPA1) activity by β-endophin in Dorsal Root Ganglia (DRG) neurons. **(A)** Activation or blockade of MOR by RvD1 (100 nM) was assessed by a Förster resonance energy transfer (FRET) assay in HEK-293 cells co-transfected with MOR and Gα subunits as indicated. Representative measurements are shown. Fluorescence intensity is given as FRET ratio normalized to baseline. DAMGO (10 μM) served as positive control. **(B)** Fura-2/AM-based single cell calcium imaging (ratio F(340/380)) in dissected, cultured DRGs of adult mice. Preincubation of the DRGs in β-endorphin (100 nM, 5 min) did not further increase intracellular calcium upon TRPA1 stimulation with allyl isothiocyanate (AITC ; 10 μM). Traces represent mean ± SD of 7–9 independent measurements per group. **(C,D)** Statistical analysis of the number of reacting cells **(C)** and the area under curve **(D)** demonstrate no significant differences between the treatment groups. (AITC: *n* = 7; pretreatment with β-endorphin: *n* = 9; mean ± SEM, student’s *t*-test, *p* < 0.05).

Because of the evidence for the involvement of opioids in RvD1- and Chem-mediated antinociception and the preference of resolvins to interact with GPCR, we used an *in vitro* model to assess MOR agonist-properties. HEK-293 cells were co-transfected with untagged MOR and a G protein FRET sensor subtype: Gαi1, Gαi2, or Gαi3, respectively (Figure 4A). The synthetic opioid peptide DAMGO, which has a high affinity to MOR, served as positive control. While DAMGO reduced the FRET ratio in all experiments, RvD1 did not alter the signal. Neither did RvD1 reduced the FRET ratio nor did it prevent DAMGO-induced lowering of FRET ratios in a Gαi subunits. Application of buffer solution washed out the compounds and restored basal values.

### Unaltered TRPA1-Induced Calcium Influx by β-endorphin

Next, we tested whether endogenous β-endorphin modulate TRPA1 activity in DRGs. Cultured adult DRG neurons from of C57BL/6 mice were loaded with Fura/2-AM, a ratiometric calcium dye. Neurons were incubated in β-endorphin before stimulation with allyl isothiocyanate (AITC), a specific TRPA1 agonist (Figure 4B). β-endorphin neither increased nor decreased the number of TRPA1-positive neurons (Figure 4C) or the area under curve (Figure 4D).

### RvD1 does Not Recruit β-Arrestin

**Figure 5:**
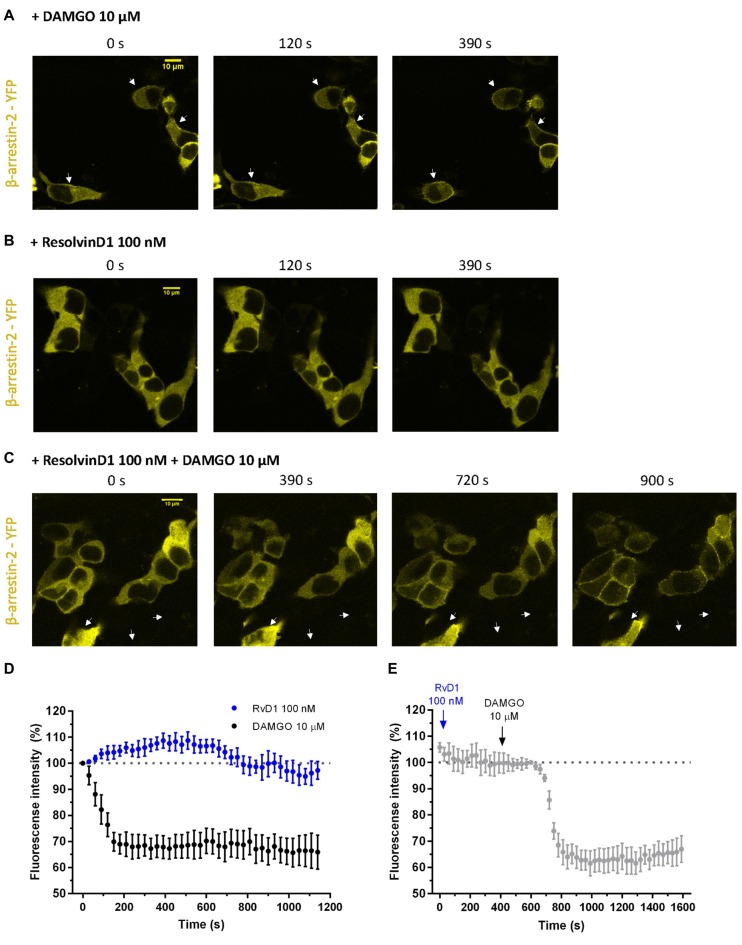
β-arrestin-2 recruitment mediated by MOP in response to DAMGO or/and ResolvinD1. Representative images from HEK-293 cells transfected with MOR-CFP, GRK2, and β-arrestin-2-yellow fluorescent protein (YFP), are shown at different time points (0, 120, 390, 720 or 900 s) upon application of the indicated concentration of ligand: 10 μM DAMGO **(A)**, 100 nM RvD1 **(B)** or 100 nM RvD1 followed by addition of 10 μM DAMGO after 450 s **(C)**. Arrows indicate β-arrestin-2 recruitment. **(D,E)** Images were quantified and corrected for photobleaching of the sample (DAMGO = 7 cells; RvD1 = 8 cells; RvD1 + DAMGO = 10 cells).

To assess whether β-arrestin is crucial for TRP channel blockade by resolvins, we transiently transfected HEK-293 cells with β-arrestin-2-YFP, GRK2 and MOR-CFP (Figure 5). FRET measurements were performed. While DAMGO induced a translocation of β-arrestin to the membrane (Figures 5A,D), RvD1 failed (Figures 5B,D). Furthermore, RvD1 did not block the DAMGO-induced β-arrestin recruitment (Figures 5C,E).

### Deficit of Endogenous Peripheral MOR Antinociception Impairs TRPA1 Antinociceptive Properties

**Figure 6:**
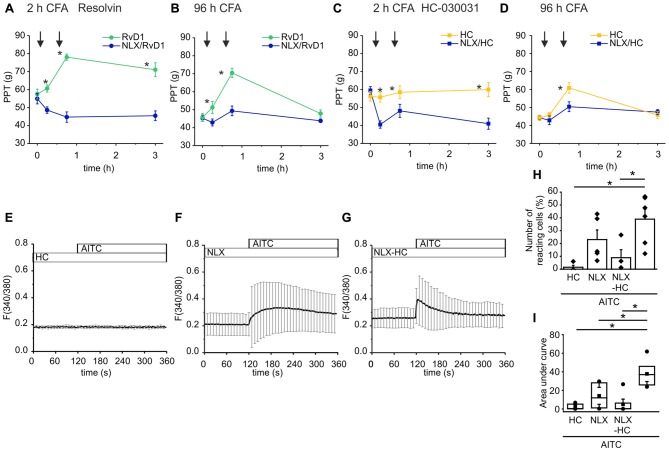
Naloxone reverses antagonism of RvD1 and HC-030031 *in vivo* and impairs TRPA1 blockade *in vitro*. **(A–D)** Measurements of PPTs (g) in the early **(A,C)** and the late phase **(B,D)** of CFA-induced inflammation. First naloxone was injected (1st arrow, blue lines) or saline (yellow/green lines). Subsequently, naloxone plus RvD1 **(A,B)** or HC-030031 **(C,D)** were injected in the corresponding naloxone group (blue lines; 2nd arrow) while only the TRPA1 antagonists RvD1 (**A,B**; green) or HC-030031 (**C,D**; yellow) were applied in the control groups. Mechanical nociceptive thresholds were obtained at indicated time points. (*n* = 5 per group, mean ± SEM; two-way ANOVA RM *post hoc* Holm-Sidak, *p < 0.05 naloxone + RvD1/HC-030031 vs. solvent + RvD1/HC-030031). **(E–G)** Representative measurements of calcium transients in DRG neurons pre-incubated for 5 min in HC-030031 (10 μM), naloxone (10 μM) or HC-030031 plus naloxone before stimulation with AITC (10 μM) at indicated time points. Mean ± S.D. **(H,I)** Statistical analysis of the number of reacting cells (%; **H**) area under curve (taken from 160 s to 340 s; **I**). AITC: *n* = 6; pretreatment with HC-030031 *n* = 4; pretreatment with naloxone: *n* = 5; pretreatment with HC-030031 plus naloxone: *n* = 7; mean ± SEM, One-way ANOVA *post hoc* Holm-Sidak, *p* < 0.05.

Endogenous opioid peptides are tonically secreted and mediate thermal antinociception in early inflammation (Rittner et al., 2009). Here, we proposed that the balance of endogenous pro- and anti-hyperalgesic mediators shapes mechanical hypersensitivity. Hence, application of antagonists affects mechanical nociceptive thresholds. In early CFA-evoked inflammation, intraplantar injection of naloxone (blue lines, 1st arrow) further lowered mechanical PPTs (Figures 6A,C). No further decrease of the nociceptive thresholds by naloxone was observed in late inflammation (Figures 6B,D). Subsequently, rats receive an intraplantar co-injection of the TRPA1 antagonist RvD1 (Figures 6A,B) or HC-030031 (Figures 6C,D) and naloxone (2nd arrow). However, the TRPA1 inhibitors failed to ameliorate hypersensitivity in presence of naloxone. Mechanical nociceptive thresholds in the naloxone group were significantly lower than in the control groups where rats only received the TRPA1 inhibitor RvD1 or HC-030031.

To mimic the reversal of TRPA1 blockade by naloxone *in vitro*, we measured calcium transients in DRG neurons upon stimulation with AITC. Pre-incubation of the neuronal cells in HC-030031 prevented the TRPA1-mediated intracellular calcium increase evoked by AITC (Figure 6E). Pre-incubation of the cells with naloxone diminished the AITC-elicited calcium transients (Figures 6G,F). The activation pattern turned out to be more in-homogenous. While in some experiments, the number of activated cells was reduced compare to stimulation with AITC (Figure 6H) the area under curve was lowered significantly (Figure 6I). On the other hand, naloxone impaired the blockade of TRPA1 by HC-030031. The AITC-induced calcium increase was restored when cells we pre-treated with HC-030031 and naloxone at the same time (Figure 6D). Since only some neuronal cells respond to the AITC stimulus, the area under curve did not increase significantly.

## Discussion

In this study, local RvD1 and Chem raise mechanical nociceptive thresholds in the early neutrophil dominated phase as well as in the late macrophage phase of CFA-induced inflammation. Local blockade of MOR or opioid peptides as well as neutrophil depletion prevent antinociception by RvD1 and Chem. Both, RvD1 and Chem do not stimulate neutrophils or macrophages to secret opioid peptides nor does RvD1 directly activate MOR *in vitro*. Therefore, we hypothesize that either an altered balance of pro- and antinociceptive mediators or intracellular pathways by interaction of GPCR and TRPA1 are responsible. *In vitro*, β-endorphin does not sensitize or desensitize TRPA1 in DRG neurons, but naloxone hampers TRPA1 blockade by HC-030031. *In vivo*, local MOR blockade further decreases mechanical nociceptive thresholds shifting the thresholds towards a further enhanced hypersensitivity. Subsequently, antinociception by RvD1 and HC-030031, the most specific TRPA1 blocker, was diminished. These findings support the hypothesis of either a dysbalance of endogenous pro- and antinociceptive mechanisms or an interaction of TRPA1 and opioid receptors.

### Antinociception by Resolvins

Up to now, most of the studies analyzed central antinociceptive effects of resolvins. For example, intrathecal application of RvD1 and RvE1 increases thermal nociceptive thresholds after CFA (Xu et al., 2010). Spinal RvD1 elicits potent antinociceptive effect in lumbar disc herniation (Liu et al., 2016), prevents long-term hypersensitivity after thoracotomy (Wang and Strichartz, 2017) as well as chronic pancreatitis-induced visceral pain (Quan-Xin et al., 2012) and 17(R)-HDoHE reverses pain behavior in two models of osteoarthritis pain (Huang et al., 2017). Less is known about peripheral effects and about mechanical hypersensitivity: intraplantar RvD1 and RvE1 reduced carrageen-induced thermal hypersensitivity and pain behavior evoked by capsaicin or AITC, respectively (Park et al., 2011b). Here, we describe peripheral antinociception by RvD1 and Chem in early and late CFA-induced inflammation on mechanical nociceptive thresholds.

Four different antinociceptive mechanisms by resolvins have been implicated: first, in the resolution phase of inflammation in the inflamed tissue, resolvins downregulate the recruitment and function of immune cells (Serhan, 2014) and thereby reduce pain. GPCR activation diminishes the secretion of pro-inflammatory cytokines and chemokines (Ji et al., 2011; Serhan, 2014; Lim et al., 2015). Low concentrations of RvD1 specifically limit the infiltration of neutrophils, polarize monocytes and increase phagocytosis of apoptotic cells by macrophages via GPR32 (Arita et al., 2007; Cash et al., 2008; Merched et al., 2008; Krishnamoorthy et al., 2010, 2012; Norling et al., 2012; Schmid et al., 2016). The function of Chem is more diverse: Chem binding to Chem23R is a potent macrophage chemoattractant protein (Hart and Greaves, 2010). On the other hand, Chem inhibits pain behavior in the second phase of the formalin test when injected intrathecally (Xu et al., 2010) or CFA-induced hypersensitivity as shown here. Rapid relief of hypersensitivity observed *in vivo* appeared to be mediated by receptor activation/inhibition independent of intercellular actions such as recruitment or activation of innate immune cells.

Second, intrathecal application of resolvins reduces spinal inflammation by inhibition of TNF-α and IL-1β, stimulation of IL-10 and TGF-β1, and attenuation of NFκB/p65 and p-ERK expression in a dose-dependent manner (Liu et al., 2016).

Third, activation of resolvin receptors inhibits TRP activity. Several groups suggest that resolvin-mediated blockade of TRP channels acts via a G-protein regulated mechanism (Choi and Hwang, 2016). Pathways include a phosphorylation cascade inhibiting the adenylate cyclase or ERK (Park et al., 2011b; Serhan, 2014; Lim et al., 2015) or β-arrestin coupled to resolvin receptors (Krishnamoorthy et al., 2010). Indeed, β-arrestin recruitment could lead to increased TRP channel sensitization or desensitization (Por et al., 2013; Rowan et al., 2014). Coupling of resolvin receptors with TRP channels in nociceptor terminals would be needed to be supported by further experimental evidence. Since ChemR23 and TRPV1 are co-expressed in small diameter DRGs, it is likely that RvE1-mediated inhibition of TRPV1 (Xu et al., 2010) arises upon ChemR23 stimulation (Jo et al., 2016). Taken together, the exact mechanism of resolvin-mediated TRP channel blockade needs further exploration.

Fourth, interactions with opioid receptors have been explored. MOR did not mediate the central antinociceptive action of RvE1, as the MOR antagonist naloxone did not block (Xu et al., 2010). In fact, RvD1 is not a MOR agonist *in vitro* because is neither activates Gi nor recruits β-arrestin directly in MOR-expressing HEK-293. So, MOR is not the GPCR for the ligand RvD1.

In our studies, we not only observed an interaction of resolvins with MOR but also TRPA1 with MOR. In recent years, protein-protein connections have been analyzed in depth supporting the complexity of the pain pathway (Rouwette et al., 2015). Protein-protein interactions include receptors, kinases or adapter proteins. For example, A-kinase anchor protein 79/150 (AKAP79/150) binds to TRPV1, Tmem100, a membrane adaptor protein, potentiates TRPA1 activity (Weng et al., 2015) and annexin A2 impairs TRPA1 availability in the plasma membrane (Avenali et al., 2014). Therefore, it is possible that MOR and TRPA1 interact. In addition, N-methyl-D-aspartate (NMDA) receptor are a possible link between opioid receptors and TRPV1 (Mao, 1999; Lee et al., 2012).

Naloxone as well as naloxone-methiodide are considered to be non-specific opioid receptor antagonists, because they can reverse antinociceptive effects of MOR, DOR and κ-opioid receptors (KOR; Labuz et al., 2007). Since the *in vivo* system is very complex, conclusions drawn from *in vitro* experiments have to be evaluated carefully. Mimicking the *in vivo* situation in *in vitro* setups is limited as recently shown by matching the data of *in vivo* and *in vitro* calcium imaging in the DRG (Emery et al., 2016). The comparison of naloxone effects *in vivo* and *in vitro* is speculative. *In vivo*, it could be suggested that inhibitory effects of endogenous opioids released from immune cells in inflammation are blocked by naloxone. In our study here, endogenous opioids seem to interact in the periphery. Naloxone prevented antinociception by RvD1, Chem or HC-030031 in CFA-induced inflammation. Thus, the endogenous pro- and anti-nociceptive balance is disturbed. In addition, we observed the reduction of inhibitory effects of TRPA1 inhibitors in the presence of naloxone in DRG neurons *in vitro*. Viewing from the pharmacological site, relatively high doses of naloxone *in vitro* (e.g., 10 μM) could also possibly directly block TRPA1.

Naloxone *per se* reverses opioid-mediated analgesia. Whether the application of naloxone aggravates pain or prevents inhibitory effects of other nociceptor blockers in the periphery, as shown here, has not been studied so far. Indeed, interactions of TRPA1 and opioid receptors have been explored before in a model of visceral pain (Pereira et al., 2013). Hypersensitivity of the abdomen after ifosfamide-induced hemorrhagic cystitis was lessened by systemic application of the TRPA1 antagonist HC-030031. These authors observed only a small and insignificant reversal of HC-030031-antinociception by naloxone. However, they used a much higher dose applied systemically (2 mg/kg i.p. vs. 0.56 ng intraplantar), which presumably blocks central opioid receptors, and a different model of pain.

### Opioid Peptide Release from Immune Cells

Opioid peptides like β-endorphin, met-enkephalin and dynorphin are secreted from neutrophils or monocytes/macrophages upon stimulation (Plein and Rittner, 2017) by Chemokines (Rittner et al., 2006), bacterial products (Rittner et al., 2009; Sauer et al., 2014) or exogenous opioids itself (Celik et al., 2016; Labuz et al., 2016). Opioid peptide release is constantly present in inflamed tissue, because intraplantar injection of anti-opioid peptide antibodies or naloxone further lower thermal nociceptive thresholds (Rittner et al., 2009). Similarly, anti-PMN serum depleting neutrophils (Rittner et al., 2006, 2009) or cytotoxic drugs like cyclophosphamide depleting immune cells further worsen mechanical hypersensitivity in rats with CFA-induced hindpaw inflammation (Sauer et al., 2014). We have extensively used peritoneal neutrophils and macrophages for *in vitro* experiments before and found matching results between *in vivo* and *in vitro* (Rittner et al., 2006, 2009; Sauer et al., 2014). To our knowledge immune cells isolated from the inflamed paw tissue have not been used for release of opioid peptides from immune cells so far. Schreiter et al. (2012) used membranes from infiltrating immune cells to analyze aminopeptidase N and neutral enkephalinase activity. Celik et al. (2016) employed immune cells isolated from the nerve to study release of opioid peptides *in vitro*. Nevertheless, based on our results, we argue that even cells from the tissue would not release opioid peptides in response to resolvin. Furthermore, we now provide evidence that local naloxone also reduces mechanical nociceptive thresholds measured by Randall-Selitto test especially in early stages of CFA-induced hindpaw inflammation. We did not observe this at later time points possibly due to more pronounced hypersensitivity and detection thresholds of the Randall-Selitto test.

Next, we proposed that RvD1 stimulates the release of opioid peptides. RvD1 augments synthesis and release of anti-inflammatory cytokines while it decreases the production of proinflammatory cytokines. RvE1 rises intracellular Ca^2+^ levels (Arita et al., 2007). We have previously shown that the Gα_i/o_-Gβγ protein-PLC-IP3 receptors-intracellular Ca^2+^ pathways regulate the opioid peptide release within the cell (Rittner et al., 2006, 2009; Sauer et al., 2014; Celik et al., 2016). Therefore, we asked whether RvD1 or Chem could stimulate release of opioid peptides via intracellular calcium increase. However, *in vitro* RvD1 was not able to stimulate the release of β-endorphin in peritoneal macrophages or neutrophils. One explanation could be that RvD1 rather decreases arachidonic acid or cAMP mediated intracellular Ca^2+^ increase (Fredman et al., 2014). Alternatively, RvD1 is not strong enough *in vitro*, since cells were preactivated by recruitment into the peritoneal cavity and with cytochalasin. Taken together, RvD1-induced opioid peptide release seems not to be a prominent mechanism *in vivo*. Instead, we provide evidence that *in vivo* blockade of MOR shifts the balance of pro-nociceptive and anti-nociceptive pathways towards enhanced hypersensitivity. Therefore, although antinociceptive TRPA1 pathways are blocked by RvD1 or specific blockers, this only reverses the intense hypersensitivity but it is not potent enough to normalize mechanical nociceptive thresholds.

### Future Clinical Implications

Resolvins are very promising candidates to treat inflammatory pain. Interestingly, these endogenous lipid derivatives can contribute to the resolution of two different pathologic processes, pain and inflammation via differential cellular targets and biological mechanisms. No adverse effects have been reported so far in *in vivo* studies. Although conceivably, premature termination of inflammation or pain could be harmful. Unfortunately, a proof of concept clarifying the relevant molecular mechanisms for pain inhibition has not been provided yet (Choi and Hwang, 2016). It remains to be deciphered how resolvins bind to the GPCR and how they transduce their signals. Further, the interaction with TRPV1 and TRPA1 as well as its interaction with MOR is so far not clear and needs further studies.

In the recent years, longer acting resolvins have been developed as promising drugs. Furthermore, diets rich in fish and fish oil as well as omega-3 polyunsaturated fatty acids might not only be protective in inflammatory conditions but also ameliorate pain. These aspects offer new perspectives towards the development of novel targeted interventions for the treatment of inflammatory, postoperative or neuropathic pain.

## Author Contributions

BO conducted calcium imaging experiments and analyzed all data. DH and MM performed the behavioral studies. CPV and CH tested the contribution of MOR. AB provided conceptual expertise and contributed to manuscript preparation. BO and HLR interpreted the results and wrote the manuscript.

## Conflict of Interest Statement

The authors declare that the research was conducted in the absence of any commercial or financial relationships that could be construed as a potential conflict of interest.

## Abbreviations

ALX/FRP2: lipoxin A (4)/Annexin-A1 receptor formyl-peptide receptor 2
CFA: complete Freund’s adjuvant
CFP: cyan fluorescent protein
Chem: chemerin
GPCR: G-protein-coupled receptor
GPR32: G-protein-coupled receptor 32
HBSS: Hank’s balanced salt solution
MOR: μ-opioid peptide receptor
PBS: phosphate buffered saline
PPT: paw pressure threshold
RvD1: resolvin D1
RvD2: resolvin D2
RvE1: resolvin E1
TRP: transient receptor potential
TRPA1, TRP: ankyrin 1
TRPV1, TRP: vanilloid 1
YFP: yellow fluorescent protein.
